# Post-weaning Environmental Enrichment in Male CD-1 Mice: Impact on Social Behaviors, Corticosterone Levels and Prefrontal Cytokine Expression in Adulthood

**DOI:** 10.3389/fnbeh.2018.00145

**Published:** 2018-07-17

**Authors:** Robyn Jane McQuaid, Roderick Dunn, Shlomit Jacobson-Pick, Hymie Anisman, Marie-Claude Audet

**Affiliations:** ^1^Institute of Mental Health Research, Royal Ottawa Mental Health Centre, Ottawa, ON, Canada; ^2^Department of Neuroscience, Carleton University, Ottawa, ON, Canada; ^3^School of Nutrition Sciences, University of Ottawa, Ottawa, ON, Canada

**Keywords:** aggression, enrichment, mice, PFC, cytokines, stress resilience

## Abstract

Environmental enrichment is typically associated with enhanced well-being, improved cognitive function and stress resilience. However, in some instances grouping adult male mice in enriched conditions promoted a stressful environment, which resulted in elevated endocrine, monoamine and inflammatory outcomes in response to subsequent stressor exposure. The current investigation examined whether raising male mice in an enriched environment (EE) would modulate social and anxiety-like behaviors in early adulthood and influence brain expression of pro-inflammatory cytokines and brain-derived neurotrophic factor (BDNF). Immediately after weaning (postnatal day [PD] 21), CD-1 male mice were housed with their siblings (3/cage) for 6 weeks in an EE or a standard (SE) environment. Body weights and aggressive interactions were monitored weekly. Social avoidance behaviors in the social interaction test and anxiety-like behaviors in the elevated-plus maze were examined in early adulthood. Ninety minutes following the behavioral tests, mice were sacrificed and a blood sample and the prefrontal cortex (PFC) were collected for the determination of plasma corticosterone levels as well as cytokine and BDNF mRNA expression. Mice raised in an EE exhibited more wounds and gained less weight than mice housed in a SE. Enriched mice also spent a greater amount of time in proximity of a social target in the social interaction test and made fewer transitions into the closed arms of the elevated-plus maze. Interestingly, the elevated plasma corticosterone and upregulated prefrontal interleukin (IL)-1β expression observed after the social interaction test among the SE mice were not apparent among those housed in an EE. Enrichment also increased prefrontal BDNF expression, especially among mice that experienced the social interaction test. These results suggest that although raising male mice in an EE may elicit aggressive interactions between sibling cage-mates (as indicated by a high number of wounds), this environment also enhances social behaviors and limits the corticosterone and cytokine impacts of mild social stressors encountered in early adulthood.

## Introduction

Environmental conditions may have a substantial influence over animal behaviors and well-being (Kentner, 2015). Enriched environments (EE) in laboratory rodents comprise housing in large cages equipped with toys and exercise items that stimulate playful behavior and physical activity. This manipulation may enhance cognitive abilities (He et al., 2017; Zeleznikow-Johnston et al., 2017) and diminish anxiety- and depressive-like behaviors (Galani et al., 2007; Nicolas et al., 2015; Reichmann et al., 2016; Aujnarain et al., 2018). Behavioral and cognitive effects of EE have been linked to increased neurogenesis and elevated expression of hippocampal brain-derived neutrophic factor (BDNF; Schloesser et al., 2010; Novkovic et al., 2015), and improved synaptic and transcriptomic capacity (Hüttenrauch et al., 2016; Zhang et al., 2018). In addition to improving psychological and physiological well-being in otherwise healthy animals, EE may limit behavioral, cognitive and biological disturbances provoked by stressful experiences. For instance, EE reduced anxiety-like behaviors and prevented cognitive impairments ordinarily elicited by acute and chronic stressors (Cordner and Tamashiro, 2016; Bahi, 2017; Marianno et al., 2017; Dandi et al., 2018). Mice housed in EE also exhibited limited corticosterone elevations and neuronal activation after stressor exposure (Branchi et al., 2013; Reichmann et al., 2013; Mesa-Gresa et al., 2016), indicating that housing conditions may modulate the impact of external stressors on hormonal and brain functions.

Although the stress buffering impacts of EE have frequently been confirmed, other reports, including from our laboratory, indicated that in some instances EE promoted territorial and aggressive behaviors that mitigated the positive effects that are often attributed to enrichment (McQuaid et al., 2012, 2013a). In this regard, male mice housed in EE in groups of 3–4 exhibited more anxiety-like behaviors and had increased corticosterone levels in response to stressors compared to their group-housed counterparts maintained in standard laboratory environments (Marashi et al., 2003; McQuaid et al., 2012, 2013a). They also displayed exaggerated monoamine and cytokine elevations ordinarily elicited by social and non-social stressors (McQuaid et al., 2012, 2013a,b). Precisely why EE elicits distress in group-housed male mice is uncertain, but it was suggested that the inherent territoriality of male mice could result in an unstable hierarchical structure and heightened aggression (Van Loo et al., 2003). In fact, although mice generally fare better in groups, laboratory confinement may inhibit natural territorial interactions that would be exhibited in the wild and thus accentuate territoriality and increase aggression (Howerton et al., 2008). Several reports have shown that the addition of stimulating objects (e.g., running wheels) to large environments among group-housed male mice promoted territorial and aggressive behaviors (Van Loo et al., 2002; Marashi et al., 2003; Howerton et al., 2008; McQuaid et al., 2012, 2013a).

Increased territoriality and aggression (and the resulting distress-related behavioral and biological outcomes) after the implementation of EE in laboratory mice have been primarily observed when the enrichment procedures were initiated in adult animals. Early environmental manipulations may dramatically influence behavioral, emotional and cognitive profiles in later life (Sánchez et al., 2001; Krugers et al., 2017). In relation to enrichment, post-weaning EE in male and female mice enhanced social preference, reduced anxiety-like behaviors, and improved spatial learning in adulthood (Hendershott et al., 2016; Aujnarain et al., 2018). As well, when housed in an EE with their siblings after weaning, male offspring born to dams stressed during pregnancy had reduced anxiety-like behaviors and limited Purkinje cell dendritic atrophy over the life course (Pascual et al., 2015). Thus, it is possible that the negative effects of social enrichment in male mice could be avoided by placing mice in EE immediately after weaning as opposed to early adulthood.

The current investigation examined the effects of a 6-week EE regimen on aggressive, social and anxiety-like behaviors, as well as on stress-induced corticosterone and brain inflammatory activation in CD-1 male mice. This outbred strain was selected as adult CD-1 male mice seem particularly prone to increased aggression in response to the addition of stimulating objects in their environment (Van Loo et al., 2002; Marashi et al., 2003; McQuaid et al., 2012). In contrast to earlier enrichment studies using this strain (Gross et al., 2011; McQuaid et al., 2012), mice were housed in sibling groups immediately after weaning in order to minimize social changes. We hypothesized that: (1) aggression levels and severity over the course of enriched housing would be comparable among SE and EE male CD-1 mice; (2) EE would increase social behaviors and reduce behaviors that reflect anxiety (Hendershott et al., 2016; Aujnarain et al., 2018); and (3) EE would limit plasma corticosterone elevations as well as brain cytokine and BDNF changes following exposure to a mild social (social interaction test) and non-social (elevated-plus maze) stressor (Rodgers et al., 1999; Koya et al., 2005; Skurlova et al., 2011).

## Materials and Methods

### Animals and Housing Conditions

Mice were bred at Carleton University from CD-1 parent stock obtained from Charles River Canada (St. Constant, QC, Canada). Immediately after weaning at postnatal day (PD) 21, male mice were housed with their siblings (3/cage) for 6 weeks in a standard (SE; *n* = 36) or an enriched (EE; *n* = 33) environment. The SE consisted of a standard polypropylene cage (27 × 21 × 14 cm) containing one cotton nestlet. The EE comprised a large polypropylene rat maternity cage (50 × 40 × 20 cm) equipped with two running wheels, one orange polypropylene shelter attached to an angled running wheel, one red polypropylene shelter, three yellow polypropylene tunnels, and two cotton nestlets, as previously described (McQuaid et al., 2012, 2013a,b). Enrichment objects were replaced with clean duplicates once a week during cage cleaning but were otherwise not manipulated. Mice were left in their respective environments undisturbed, except during cage cleaning (once a week for EE mice and twice a week for SE mice due to the smaller dimensions of the housing environment) and tail marking (three times/week for both EE and SE mice; at the base of the tail, using a color-coding system with non-toxic markers). All mice were kept in the same temperature (21°C) and humidity (63%) controlled room, given free access to food and tap water, and maintained on a 12-h light/dark cycle, with lights on from 08:00 h to 20:00 h. All experimental procedures were approved by the Carleton University Animal Care Committee and met the guidelines set out by the Canadian Council on Animal Care.

### Weighing and Aggression Scoring

Mice were weighed immediately after weaning (before being placed into their respective environments) and then once a week during the 6-week SE and EE housing. The percentages of weight change from weaning were determined at the end of each week ([Weight at Week X − Weight at weaning]/Weight at weaning). Home-cage interactions were videotaped for 30 min at the same time of day (between 10:00 h and 11:00 h) every week. As hierarchical reorganization is highest after cage cleaning (Van Loo et al., 2000), videotaping was carried out immediately after mice were placed into their clean cages. Experimenters blind to the housing conditions subsequently scored, manually, aggressive behaviors from videotapes. These included Attacking (biting or attempting to bite, sometimes accompanied by tail rattling), Aggressive chasing (rushing or jumping at another mouse), Aggressive grooming (vigorous sniffing or licking), and Fighting (aggressive wrestling between two or more mice). The presence of wounds was also examined after cage cleaning and tail marking sessions as an indirect measure of aggression.

### Behavioral Tests

After being housed in a SE or an EE for 6 weeks, mice were brought to their respective testing rooms 24 h prior to the behavioral test sessions to acclimate to the new laboratory environments. Behavioral testing took place between 08:00 h and 13:00 h to minimize effects related to diurnal factors. Mice within a same cage were randomly assigned to the social interaction test or the elevated-plus maze test (which also served as mild social or non-social stressors, respectively) or were not manipulated (no stressor condition). After the behavioral tests, mice were returned to their respective environments for 90 min, after which they were sacrificed and blood and brain tissue were collected.

#### Social Interaction Test

**Scheme 1:**
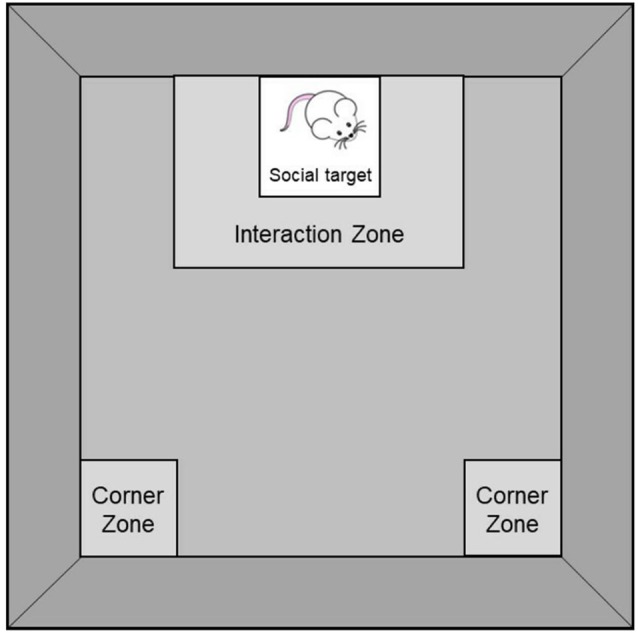
Social Interaction Test comprising an 8 × 8 cm Interaction Zone surrounding a wire-mesh enclosure (10 × 10 × 30 cm; where a social target is introduced during the second session of the test) and two 8 × 8 cm Corner Zones opposing the wire-mesh enclosure.

Social behaviors were assessed in the social interaction test (see Scheme 1). As previously described (Berton et al., 2006; Szyszkowicz et al., 2017), mice were placed, individually, into a black Plexiglas open field (42 × 42 × 60 cm) that contained a wire-mesh enclosure (10 × 10 × 30 cm) for two sessions of 150 s each, under red-light conditions. During the first session, the enclosure was empty whereas during the second session, a 6-month-old male CD-1 retired breeder (social target) was present in the enclosure. Between the two sessions, mice were placed into an empty cage with bedding for about 60 s during which open field surfaces were cleaned with a 70% ethanol solution and wire-mesh enclosures were switched. The two sessions were videotaped with an infra-red camera and movements were subsequently scored manually by an experimenter blind to the housing conditions. Time spent in an 8-cm corridor surrounding the enclosure (Interaction Zone) and in the 8 × 8 cm corner areas opposing the wire-mesh enclosure (Corner Zones) were used as measures of social avoidance. Because chronic social defeat, which is typically used to elicit social avoidance in this test (Berton et al., 2006; Szyszkowicz et al., 2017), was not used in the current experiment, a social interaction ratio (which classifies mice as susceptible or resilient to the effects of social stress) was not established.

#### Elevated-Plus Maze

Anxiety-like behaviors were assessed in the elevated-plus maze, which was composed of two opposing open arms (50 × 10 cm) and two opposing closed arms (50 × 10 cm; with 21 cm-high walls) made of black Plexiglas. The apparatus was elevated 30 cm above a stable surface, in a dimly lit room (approximately 50 lux). Mice were individually placed at the extremity of a closed arm of the maze (facing away from the center) and their behaviors were recorded for 5 min by a ceiling-mounted video camera. Latency to enter an open arm as well as time spent in and number of entries to (defined by all four paws being in an arm) open and closed arms were subsequently scored manually by an experimenter blind to the housing conditions.

### Blood and Brain Collection

Mice were euthanized by rapid decapitation 90 min after the social interaction test or the elevated-plus maze test (which served as mild social and non-social stressors, respectively), or at corresponding time for mice that were not manipulated (non-stressed mice). Trunk blood was immediately collected in tubes containing 10 μg of EDTA, centrifuged for 8 min at 3600 RPM, and the plasma was aliquoted and stored at −80°C for subsequent determination of corticosterone levels. Brains were rapidly removed and placed on a stainless steel brain matrix (2.5 × 3.75 × 2.0 cm) positioned on a block of ice. The matrix had a series of slots spaced approximately 500 μm apart that guided razor blades to provide coronal brain sections. Once the brains were sliced, a tissue section from the prefrontal cortex (PFC) was collected based on the mouse atlas of Franklin and Paxinos (1997), placed immediately in nuclease-free tubes positioned on dry ice, and stored at −80°C for subsequent determination of the mRNA expression of the pro-inflammatory cytokines IL-6, IL-1β and tumor necrosis factor (TNF)-α and of the neurotrophin BDNF. The PFC as the brain region of interest and IL-1β, IL-6 and TNF-α as the pro-inflammatory cytokines of interest were selected based on our previous findings that male CD-1 mice had altered prefrontal mRNA expression of these cytokines after acute and chronic exposure to social stressors (see Audet et al., 2010, 2011).

### Plasma Corticosterone Determination

A commercial radioimmunoassay kit (ICN Biomedicals Inc., Costa Mesa, CA, USA) was used to determine plasma corticosterone concentrations (in duplicate). The assay was conducted in a single run to prevent inter-assay variability. The intra-assay variability was less than 10%. The assay sensitivity was 1.7 ng/ml.

### Reverse Transcription-Quantitative Polymerase Chain Reaction Analysis (RT-qPCR)

Brain sections were homogenized using Trizol and total brain RNA was isolated according to the manufacturer’s instructions (Invitrogen, Burlington, ON, Canada). RNA yields and purity were tested using a NanoDrop 2000 (Thermo Fisher Scientific). Only RNA samples with purity ratios 260/280 and 260/230 between 1.90 and 2.10 were included and thus the N and df associated with prefrontal gene expression differed from behavioral and corticosterone outcomes. The total RNA was then reverse-transcribed using Superscript II reverse transcriptase (Invitrogen, Burlington, ON, Canada). The resulting cDNA aliquots were analyzed in simultaneous quantitative polymerase chain reactions (qPCR) using SYBR green detection and a MyiQ2 Real-Time PCR Detection System (Bio-Rad, Canada). All designed PCR primer pairs generated amplicons between 129 base pairs and 200 base pairs. Amplicon identity was verified by restriction analysis. Primer efficiency was measured from the slope relation between absolute copy number of RNA quantity and the cycle threshold using the Bio-Rad IQ5 version 2.0 software (Bio-Rad, Canada). All primer pairs had a minimum of 90% efficiency.

Primers that amplify glyceraldehyde-3-phosphate dehydrogenase (GAPDH) and synaptophysin were used as reference genes. The expression of each gene of interest within the PFC was normalized by subtracting the quantification cycle (C_q_) of the two reference genes averaged from the gene of interest C_q_ (ΔC_q_). The 2^−ΔΔC_q_^ method (Livak and Schmittgen, 2001; Schmittgen and Livak, 2008) was used to convert ΔC_q_ values to mRNA fold changes relative to the SE/No stressor group (calibrator). Primer sequences used were as follows: Mus GAPDH, F: 5′-AAA TGG TGA AGG TCG GTG TG-3′, R: 5′-GAA TTT GCC GTG AGT GGA GT-3′; Mus Synaptophysin, F: 5′-GGA CGT GGT GAA TCA GCT GG-3′, R: 5′-GGC GAA GAT GGC AAA GAC C-3′; Mus IL-1β, F: 5′-TGT CTG AAG CAG CTA TGG CAA C-3′, R: 5′-CTG CCT GAA GCT CTT GTT GAT G-3′; Mus IL-6, F: 5′-TTC TTG GGA CTG ATG CTG GTG-3′, R: 5′-CAG AAT TGC CAT TGC ACA ACT C-3′. Mus TNF-α, F: 5′-CTC AGC CTC TTC TCA TTC CTG C-3′, R: 5′-GGC CAT AGA ACT GAT GAG AGG G-3′. Mus BDNF, F: 5′-GTC TCC AGG ACA GCA AAG CCA C-3′, R: 5′-CCT TGT CCG TGG ACG TTT ACT TC-3′.

### Statistical Analyses

Percentage of weight change from weaning to each of Weeks 1–6 as well as aggression scores and behaviors in the elevated-plus maze test were analyzed using a one-way analysis of variance (ANOVA) with Housing serving as the between-groups factor (SE vs. EE). The actual weight over the course the 6-week housing period was analyzed using a 2 (Housing) × 6 (Week: Weeks 1–6) mixed measures ANOVA with repeated measures on the within-groups factor comprising the 6 weeks. Behaviors in the social interaction test were analyzed using a 2 (Housing) × 2 (Session: Absence vs. Presence of CD-1 retired breeder) mixed measures ANOVA with repeated measures on the within-groups factor comprising the two test sessions. Plasma corticosterone concentrations and fold changes in prefrontal mRNA expression of pro-inflammatory cytokines and BDNF were analyzed using a 2 (Housing) × 3 (Stressor: No Stressor, Social Stressor [social interaction test] and Non-Social Stressor [elevated-plus maze]) between-groups ANOVA. Follow-up comparisons comprised *t*-tests with a Bonferroni correction to maintain the alpha level at 0.05.

## Results

### Body Weights

**Figure 1:**
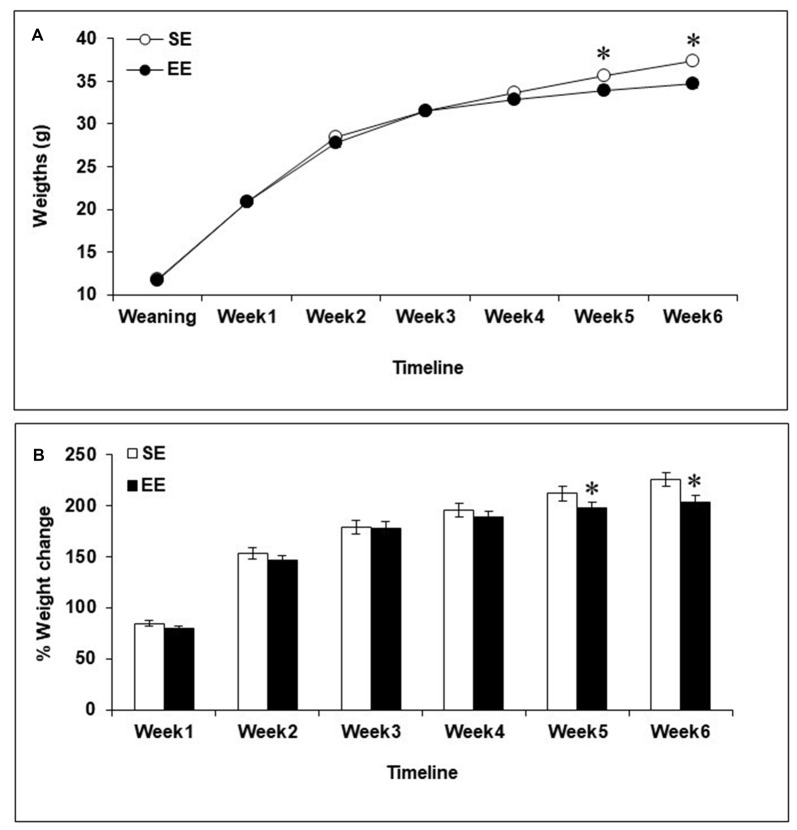
Body weights among mice housed in a standard environment (SE) or an enriched environment (EE) during 6 weeks. **(A)** Actual weights increased over the course of the 6-week housing in both SE and EE mice, but EE mice weighed less than SE mice during Weeks 5 and 6. **(B)** Mice housed in EE gained as much weight as mice housed in SE at Weeks 1–4 but at Weeks 5 and 6, percentages of weight change from weaning were reduced in EE mice compared to SE mice. *p < 0.05 relative to mice housed in SE. Data represents means ± SEM (mice housed in SE, *n* = 33; mice housed in EE: *n* = 36).

Body weights increased during the 6-week housing period, *F*_(6,402)_ = 2226.00, *p* < 0.0001, and varied as a function of the interaction between Housing and Week, *F*_(6,402)_ = 5.78, *p* < 0.0001. As shown in Figure 1A, the analysis of the simple effects comprising the Housing × Week interaction confirmed that SE and EE mice had comparable weights during Weeks 1–4, but EE mice were lighter than SE mice during Weeks 5 and 6 (*p*’s < 0.05). Percentages of weight change (i.e., weight lost or gained) were also affected by Housing during Weeks 5 and 6, *F*’s_(1,67)_ = 3.49 and 5.43, *p*’s < 0.05, with EE mice exhibiting slight, but significantly reduced weight gain compared to SE mice (Figure 1B).

### Aggression

For ethical reasons, mice that were excessively aggressive (and thus could potentially have elicited serious injuries to their cage-mates) were removed from their cages and from the study. By the end of Week 5, three EE mice had been removed, all from separate cages, but no SE mice had to be eliminated. Table 1 displays the average number of aggressive encounters (total aggressions and specific types of aggressive behaviors), as well as the average number of wounds over the 6-week housing period in EE and SE mice. As depicted in Table 1, Fights, *F*_(1,10)_ = 3.54, *p* = 0.089, were marginally more frequent among SE mice than EE mice, but this difference did not reach significance. As well, aggressive chases appear to be more frequent among SE mice, but this was exceptionally variable over the 6-week housing and the difference observed did not approach significance, *F*_(1,10)_ = 1.02, *p* = 0.40. In contrast, EE mice had more wounds than SE mice, *F*_(1,10)_ = 4.62, *p* < 0.05. Finally, the total number of aggressive encounters as well as frequencies of attacks and of aggressive grooming bouts were comparable between EE and SE mice, *F*’s_(1,10)_ = 0.07, 0.09 and 0.17, respectively.

**Table 1:** Mean ± SEM of home-cage aggressive behaviors over the 6-week housing period among mice housed in a standard environment (SE) or an enriched environment (EE).

	Total aggressive encounters	Attacks	Aggressive chases	Aggressive grooming	Fights	Wounds
SE	49.50 ± 8.43	9.67 ± 1.93	23.83 ± 4.07	11.50 ± 1.95	4.83 ± 0.84	0.50 ± 0.20
EE	41.83 ± 8.22	11.83 ± 2.26	12.50 ± 2.11	15.67 ± 3.61	0.83 ± 0.22	6.67 ± 1.15*

*p < 0.05 relative to mice housed in SE. Mice housed in SE: n = 33; mice housed in EE: n = 36. Scores were averaged per week.

### Social Interaction Test

**Figure 2:**
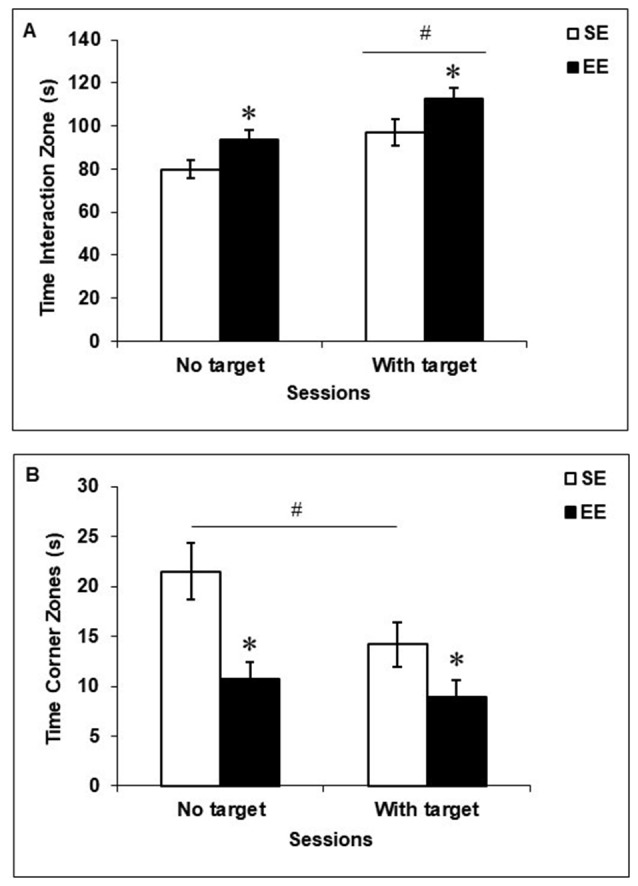
Social behaviors in the social interaction test in mice housed for 6 weeks in a SE or an EE. **(A)** EE mice spent more time in the Interaction Zone than SE mice, irrespective of whether a mouse target was present in the enclosure (*p < 0.05 relative to mice housed in SE). Both SE and EE mice spent more time in the Interaction Zone when a mouse target was present (^#^*p* < 0.001 relative to the absence of a mouse target). **(B)** Mice housed in EE spent less time in the Corner Zones than SE mice, irrespective of whether a mouse target was present in the enclosure (*p < 0.005 relative to mice housed in SE). Only SE mice spent less time in the Corner Zones in the presence of a mouse target (^#^*p* < 0.05 relative to the absence of a mouse target). Data represents means ± SEM (mice housed in SE: *n* = 12; mice housed in EE: *n* = 8).

As shown in Figure 2, EE mice spent more time in the Interaction Zone, *F*_(1,19)_ = 6.46, *p* < 0.05 (Figure 2A) and less time in the Corner Zones, *F*_(1,19)_ = 12.35, *p* < 0.005 (Figure 2B), than did SE mice, regardless of whether a social target (CD-1 retired breeder) was present in the testing environment. When a social target was introduced, both SE and EE mice spent more time in the Interaction Zone, *F*_(1,19)_ = 16.72, *p* < 0.001, but only SE mice spent less time in the Corner Zones (*p* < 0.05) compared to when the social target was absent.

### Elevated-Plus Maze Test

**Figure 3:**
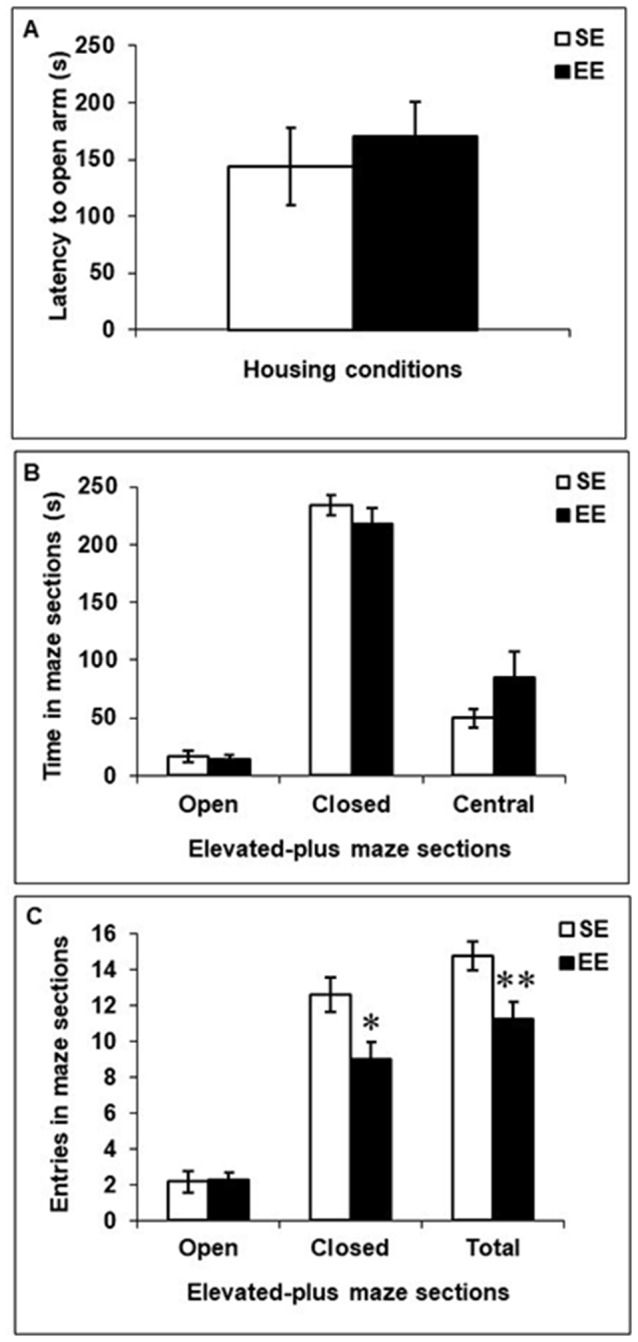
Anxiety-like behaviors in the elevated-plus maze in mice that had been housed for 6 weeks in a SE or an EE. **(A,B)** Latency to enter an open arm as well as time spent in open arms, closed arms, and the central platform of the maze were comparable between EE and SE mice. **(C)** Mice housed in EE made fewer entries in the closed arms of the maze and fewer transitions between the different arms of the maze compared to SE mice. *p < 0.05 and **p < 0.01 relative to mice housed in SE. Data represents means ± SEM (mice house in SE: *n* = 12; mice housed in EE: *n* = 8).

Figure 3 depicts behaviors in the elevated-plus maze among SE and EE mice. EE and SE mice did not differ in their latencies to enter an open arm, *F*_(1,18)_ = 0.29 (Figure 3A), the time spent in the open arms, closed arms, or central platform, *F*’s_(1,18)_ = 0.10, 1.06 and 2.98, respectively (Figure 3B), or the number of entries into open arms, *F*_(1,18)_ = 0.01 (Figure 3C). However, mice housed in EE made fewer entries into closed arms and fewer transitions between arms (open and closed combined) than did SE mice, *F*’s_(1,18)_ = 6.42 and 7.47, *p*’s < 0.05 and 0.01, respectively (Figure 3C).

### Plasma Corticosterone Levels

**Figure 4:**
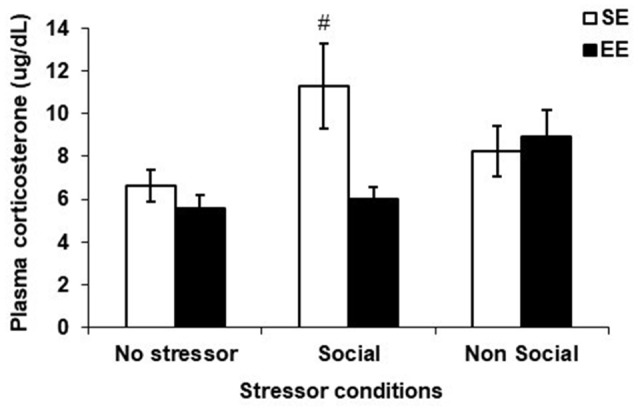
Plasma corticosterone levels collected 90 min after the social interaction test (which acted as a Social Stressor), the elevated-plus maze (which acted as a Non-Social Stressor) or a control condition (No Stressor) among mice housed for 6 weeks in a SE or an EE. Mice housed in SE had increased plasma corticosterone levels after the Social Stressor but not the Non-Social Stressor. These corticosterone elevations were not apparent in mice housed in EE. ^#^*p* < 0.05 relative to SE mice in the No Stressor condition. Data represents means ± SEM (SE mice/No stressor: *n* = 12; SE mice/Social: *n* = 12; SE mice/Non-Social: *n* = 12; EE mice/No Stressor: *n* = 11; EE mice/Social: *n* = 8; EE mice/Non-Social: *n* = 8).

Figure 4 shows the plasma corticosterone levels among SE and EE mice exposed to the social interaction test, the elevated-plus maze test or those that were left undisturbed (no stressor). Although the Housing, *F*_(1,58)_ = 3.47, *p* = 0.068, Stressor, *F*_(2,58)_ = 2.85, *p* = 0.066, and the interaction Housing × Stressor, *F*_(2,58)_ = 2.91, *p* = 0.063, did not reach significance, based on the *a priori* prediction that SE and EE mice would exhibit different corticosterone patterns after a mild social and non-social stressor, analyses of the simple effects comprising the Housing × Stressor interaction were conducted. These analyses confirmed that plasma corticosterone levels were increased 90 min after the social interaction test among SE mice (*p* < 0.05), which was not apparent in EE mice.

### Prefrontal Expression of Pro-inflammatory Cytokines and BDNF

**Figure 5:**
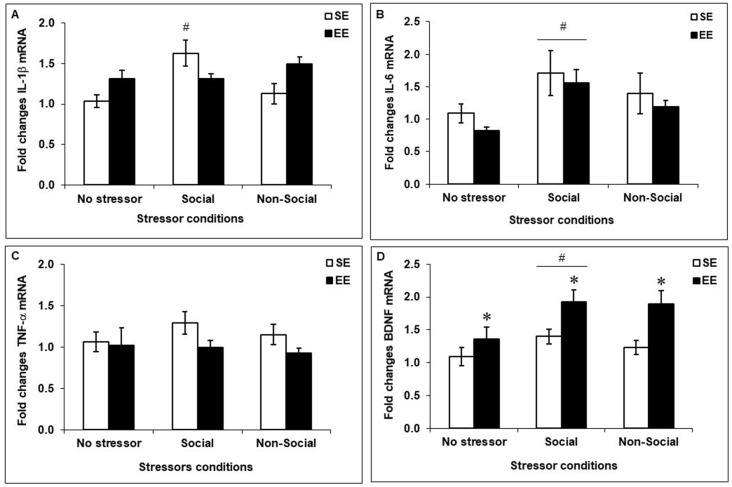
Fold change in prefrontal mRNA expression of pro-inflammatory cytokines and BDNF assessed 90 min after the social interaction test (which acted as a Social Stressor), the elevated-plus maze (which acted as a Non-Social Stressor) or a control condition (No Stressor) among mice housed for 6 weeks in a SE or an EE. **(A,B)** Mice housed in SE had increased prefrontal IL-1β and IL-6 after the Social Stressor but not the Non-Social Stressor (^#^*p*’s < 0.01 and 0.05, relative to mice in the No Stressor condition). Whereas the IL-1β increases were prevented in mice housed in EE, the IL-6 expression remained elevated. **(C)** A main effect of Housing was observed with respect to prefrontal TNF-α expression, with the cytokine being reduced in EE mice (*p* < 0.05). **(D)** Mice housed in EE had increased prefrontal BDNF expression compared to SE mice (*p < 0.005 relative to mice housed in SE). As well, BDNF expression was upregulated after the Social Stressor compared to the No Stressor condition (^#^*p* < 0.05 relative to mice in the No Stressor condition). Data represents means ± SEM (SE mice/No stressor: *n* = 6; SE mice/Social: *n* = 10; SE mice/Non-Social: *n* = 11; EE mice/No Stressor: *n* = 6; EE mice/Social: *n* = 9; EE mice/Non-Social: *n* = 5).

Fold changes in mRNA expression of the pro-inflammatory cytokines IL-1β, IL-6 and TNF-α as well as of the neurotrophin BDNF among SE and EE mice exposed to the social interaction test, the elevated-plus maze test, or left undisturbed are shown in Figure 5. Stressors affected prefrontal expression of IL-1β, *F*_(2,41)_ = 4.39, *p* < 0.05, whereas Housing did not, *F*_(1,41)_ < 1. Although the interaction between Housing and Stressor did not reach significance, *F*_(2,41)_ = 2.68, *p* = 0.08, based on the *a priori* prediction that SE and EE mice would exhibit different cytokine patterns after a mild social and non social stressor, analyses of the simple effects comprising the Housing × Stressor interaction were conducted. These analyses confirmed that, similar to corticosterone variations, PFC IL-1β expression was increased after the social interaction test in SE mice compared to non-stressed mice and mice exposed to the elevated-plus maze test (*p*’s < 0.01 and 0.005), but this effect was not apparent among EE mice. Following stressor exposure, expression of IL-6 was elevated, *F*_(2,41)_ = 3.75, *p* < 0.05, irrespective of whether mice had been housed in SE or EE. Follow-up tests confirmed that IL-6 expression was increased after the social interaction test (*p* < 0.05), but not the elevated-plus maze test (*p* = 0.25) among both SE and EE mice. In contrast, prefrontal TNF-α expression was affected by Housing, *F*_(1,41)_ = 4.53, *p* < 0.05, but was not affected by the stressors, *F*_(2,41)_ = 1.46. Specifically, TNF-α expression was reduced in EE mice (*p* < 0.05), irrespective of whether they had been exposed to the mild stressors. Finally, prefrontal BDNF expression was modulated by both the Housing, *F*_(1,41)_ = 11.27, *p* < 0.005, and the Stressors, *F*_(2,41)_ = 3.57, *p* < 0.05. Follow-up tests confirmed that the neurotrophin was increased in EE mice compared to SE mice (*p* < 0.005) as well as in mice that had experienced the social interaction test compared to those that were not stressed (*p* < 0.05).

## Discussion

In the current investigation, male CD-1 mice housed in enrichment gained less weight, displayed increased social behaviors, had limited corticosterone and prefrontal IL-1β elevations in response to a mild social stressor, and exhibited reduced TNF-α and increased BDNF expression within the PFC in early adulthood. The current paradigm housed mice together with littermates immediately after weaning, and it appears that enrichment in this instance had overall beneficial actions. Mice housed in EE displayed more wounds than SE mice over the 6-week housing period and as indicated earlier, three mice in the EE condition were removed from the study because of their excessive aggressiveness (this was required for ethical reasons). Yet, a tendency was noted for SE mice to fight more often than EE mice. These findings contrast with our previous report that male CD-1 mice placed in EE as adults displayed very aggressive behaviors towards their cage mates and showed sensitized biological processes compared to SE counterparts when placed in a novel environment (McQuaid et al., 2012). Essentially enrichment in this case had been ineffective in limiting stress responses, but instead appeared to have increased vulnerability to stressor-related outcomes. These discrepant findings raise the possibility that the timing at which male CD-1 mice are placed in enrichment is an important factor to consider for territoriality, aggression and subsequent distress to be diminished. This said, although mice housed in EE did not exhibit particularly aggressive behaviors during the 30-min weekly videotaped sessions, the possibility of agonistic encounters occurring in these mice cannot be entirely excluded, especially as they had more wounds than SE mice. Indeed, mice housed in EE were found to weigh less than SE mice during the last 2 weeks of housing, which might stem from increased distress among these mice (resulting from injurious aggression), as we previously observed (McQuaid et al., 2013a). However, the high levels of activity observed in the enrichment cages (due to access to running wheels) could also have contributed to the lower weights among EE mice, an effect found in other enrichment studies (Mesa-Gresa et al., 2016).

In the social interaction test, both SE and EE mice spent more time in proximity of a cage enclosure comprising a retired breeder (used as a social target), indicating that they were both attracted by social novelty, which is typically observed in non-stressed mice (Berton et al., 2006; Yu et al., 2011; Aujnarain et al., 2018). Importantly, mice housed in EE spent more time around the cage enclosure than SE mice, irrespective of whether a social target was present, suggesting that post-weaning enrichment may have reduced the fear of novelty (be it social or not) and/or increased the natural interest of mice for complex stimuli (e.g., a wire cage or another mouse). In line with this view, previous studies reported increased excitement and motivation to explore novel stimuli among EE-housed rodents (Roy et al., 2001; Larsson et al., 2002). Considering that mice chronically exposed to severe social stressors (e.g., social defeat) typically exhibit pronounced social avoidance in the social interaction test (Berton et al., 2006; Szyszkowicz et al., 2017), the current findings also confirm that when enrichment is provided immediately after weaning, the environment does not act as a social stressor as we have previously found when EE began during adulthood (McQuaid et al., 2012, 2013a,b), but instead enhanced sociability, as previously reported by others (Aujnarain et al., 2018).

The absence of an anxiolytic effect of enrichment in the elevated-plus maze contrasts with several reports showing a reduction of anxiety-like behaviors in the open-field test and the elevated-plus maze in male mice housed in EE from weaning onward (Chapillon et al., 1999; Aujnarain et al., 2018; Dandi et al., 2018). The reason for this discrepancy is not entirely clear, although it might be related to differences in the enrichment procedure used (e.g., dimensions of the enrichment cage, number of mice per cage, different enrichment objects and different disposition) and/or the time at which anxiety-like behaviors were assessed (e.g., diurnal vs. nocturnal phase). The current findings also differ from our previous results showing increased anxiety-like behaviors in the elevated-plus maze among male CD-1 mice housed in EE in adulthood (McQuaid et al., 2012) and again, indicate that the developmental period in which male CD-1 mice are introduced to enrichment may be important in determining whether protective or detrimental effects would be apparent with respect to emotionality. Although open arm activity was not influenced by the housing conditions, EE mice made fewer total arm entries compared to SE mice. This may simply reflect reduced activity levels, as previously demonstrated (Aujnarain et al., 2018), although this effect may also be related to the smaller number of closed arm entries (and perhaps the tendency to spend more time in the center of the maze) among EE mice.

Plasma corticosterone levels in SE mice were increased 90 min after a social interaction test, but not after the elevated-plus maze. Importantly, these elevations were not apparent in EE mice, indicating that enrichment buffered the corticosterone response to the mild social stressor. This stress buffering effect of enrichment on corticosterone levels is consistent with previous reports (Ravenelle et al., 2013; Dandi et al., 2018), suggesting that the EE might have enhanced the ability to cope with the stress of a novel social situation. Alternatively, as corticosterone levels were measured 90 min after the social interaction test, it is also possible that EE mice displayed corticosterone elevations after the test, but that levels returned to baseline more quickly as a result of the protective effects of EE. We previously reported corticosterone increases following a novel cage stressor in EE mice but not in SE mice (McQuaid et al., 2012). In this study, unrelated CD-1 mice were placed in EE in adulthood, which appeared to have promoted aggressive interactions and created a stressful environment. It is possible that in the current study, relatedness and post-weaning introduction to EE among the male CD-1 mice prevented sensitization of the corticosterone response.

Prefrontal expression of the pro-inflammatory cytokines IL-1β and IL-6 was increased after the social interaction test but not after the elevated-plus maze, indicating that stressors of a social nature may be more potent in eliciting brain inflammatory activation, as previously reported (Audet et al., 2010, 2011), or that the social interaction test was simply more stressful than placement on an elevated-plus maze. Similar to plasma corticosterone patterns, enrichment prevented the IL-1β elevations, an effect that was not apparent with regard to IL-6, which remained elevated in EE mice. Why these two cytokines in the PFC were differentially modulated by enrichment is not certain. Similar to the current findings, prefrontal IL-6 increases elicited by repeated social defeat in adult male mice remained elevated in mice that had been housed in EE, but in the hippocampus, the cytokine increases were more pronounced (McQuaid et al., 2013b), suggesting that the brain cytokine effects of enrichment may be cytokine- and region-specific. In line with this view, hippocampal elevations of IL-1β in a mouse model of influenza were limited in mice that had been housed in an EE, whereas IL-6 increases in influenza-exposed mice were not influenced by enrichment (Jurgens and Johnson, 2012). As well, hippocampal IL-1β and TNF-α elevations in rats that had undergone surgery (Briones et al., 2013; Kawano et al., 2015) or that were treated with the bacterial endotoxin lipopolysaccharide (Williamson et al., 2012), were attenuated by enrichment. Interestingly, enrichment in the current investigation reduced TNF-α expression in the PFC, but this effect appeared to be most prominent among mice exposed to the social interaction test and the elevated-plus maze. Finally, enrichment increased prefrontal BDNF expression, irrespective of whether mice were tested in the social interaction test or the elevated-plus maze, although this effect was more pronounced in mice exposed to the social stressor (which may have acted as a social arousing stimulus). Enrichment-induced BDNF elevations are typically observed in the hippocampus (e.g., Jurgens and Johnson, 2012; Novkovic et al., 2015), and the current findings suggest that it might also be the case in other stress-related brain regions.

The current investigation provides insights concerning the effects of enrichment on stress reactivity, with a focus on the importance of this procedure being initiated relatively early in development. Although EE mice exhibited more wounds, and thus potentially were involved in more injurious fighting, it appears that the environmental conditions were not sufficiently distressing to alter social and anxiety-like behaviors or to sensitize the corticosterone and cytokine effects of a mild social stressor. In fact, enrichment provided immediately after weaning appears to be beneficial, as reflected by the enhanced sociability and attenuated corticosterone and IL-1β increases induced by a mild social stressor in EE mice. Early implementation of enrichment, long-term cohabitation, and familiarity between mice may thus be key factors to consider in enrichment studies in male mice in order to promote stress resilience. Despite these encouraging findings, it needs to be underscored that the present investigation involved a relatively small number of mice. Moreover, while fighting was reduced by the EE procedure used in the current study, some mice exhibited particularly pronounced aggressive behaviors. The individual factors responsible for this are uncertain, but determining what might produce such outcomes may be important in the understanding of aggressive behaviors.

## Author Contributions

M-CA, RJM and HA designed the experiment. M-CA, RJM, RD and SJ-P conducted the experiment. M-CA, RJM and SJ-P analyzed the data. M-CA, RJM, RD and HA wrote the manuscript.

## Conflict of Interest Statement

The authors declare that the research was conducted in the absence of any commercial or financial relationships that could be construed as a potential conflict of interest.
